# Psychometric assessment of the Chinese version of the Problems and Needs in Palliative Care questionnaire-short version in advanced cancer patients

**DOI:** 10.1186/s12904-019-0450-5

**Published:** 2019-08-06

**Authors:** Tao Wang, Alex Molassiotis, Betty Pui Man Chung, Jing-Yu Tan

**Affiliations:** 1 School of Nursing, The Hong Kong Polytechnic University, Hung Hom, Kowloon, Hong Kong; 2 College of Nursing and Midwifery, Charles Darwin University, Darwin, NT Australia

**Keywords:** Palliative care, Needs, Cancer, Psychometrics, Reliability, Validity

## Abstract

**Background:**

To determine the validity, reliability and acceptability of the Mandarin Chinese version of the Problems and Needs in Palliative Care questionnaire-short version (PNPC-sv) for measuring problems and palliative care needs among patients with advanced cancer.

**Methods:**

This was a validation study using a forward- and backward- translation procedure, a panel of experts and a cross-sectional study design. The Mandarin Chinese version of the PNPC-sv was translated by four independent translators. The translated Chinese version was further reviewed by an expert panel to identify its content validity. A pilot test was conducted in 10 advanced cancer patients to preliminarily assess the face validity, readability and clarity of the pre-final version of the PNPC-sv. 178 patients with advanced cancer, regardless of their gender and types of cancer diagnosis, were further recruited through a convenience sampling from three tertiary hospitals in China to assess the psychometric properties of the PNPC-sv Mandarin Chinese version. Content validity was measured using the content validity index (CVI). Construct validity was estimated via confirmatory factor analysis and the contrasted groups approach. Concurrent validity was identified by analysing the correlations between the EORTC Quality-of-Life Questionnaire-Core 30 (EORTC QLQ-C30) and the PNPC-sv. Reliability of the PNPC-sv was examined with the internal consistency reliability and item-to-total correlations. Several closed-ended and open-ended questions were designed to explore its acceptability.

**Results:**

174 patients completed the questionnaires. High content and face validity were determined after the two rounds of assessment with the expert panel and the patients. An excellent CVI of 1.0 was achieved and patients rated the PNPC-sv as a useful instrument for assessing their problems and needs (mean score = 7.99, 0–10 scale) and reported the items were not particularly sensitive and easy to understand. The majority of the fit indexes meet the critical criteria, with the Chi-square divided by degrees of freedom (x^2^/df) being 1.58 and 2.05, and the root mean squared error of approximation (RMSEA) being 0.06 and 0.07 for the *problem* part and *need-for-care* part, respectively. In relation to the contrasted groups analysis, it clearly discriminated the differences on the sub-scores of Activities of Daily Life (ADL), spiritual and psychological problems and needs between male and female patients; ADL, physical, social and financial problems and needs between age groups; and autonomic problems and needs between patients with different cancer stages. Statistically significant correlations (*p* < 0.05) were detected between the PNPC-sv and the EORTC QLQ-C30 in the majority of the sub-scores (positive correlations) and total scores (negative correlations). The Cronbach’s alpha of the total scale was 0.88 and 0.91 for the *problem* part and *need-for-care* part, respectively. The Cronbach’s alpha of the subscales were generally above 0.70. Item-to-total correlations were generally acceptable, with the majority of the values being above 0.40. The PNPC-sv questionnaire was reported to be convenient and easy to understand, and the average time for completing was 11 min.

**Conclusions:**

The Mandarin Chinese version of the PNPC-sv is a valid, reliable and user-friendly instrument for measuring problems and palliative care needs among patients with advanced cancer. Further research is needed to further examine its psychometric properties particular internal structure in a larger patient sample.

## Background

Experiences and symptom distress of cancer patients vary across their illness trajectory [1]. Cancer patients at an advanced stage usually encounter more difficulties in optimising their well-being than those at an early stage, which subsequently contribute to a poor quality of life and an increasing demand of care needs [1, 2]. Quality of life is the patients’ subjective view of their overall life satisfaction and their sense of well-being, which involves multidimensional components including physical, psychological, social, etc. [3]. According to the definition proposed by the World Health Organization [4], palliative care is an approach that aims to optimize patients’ well-being and improve quality of life through addressing their multidimensional problems and needs. A recent systematic review conducted by our group highlighted that advanced cancer patients had a wide range of palliative care needs and their needs were somewhat context-bound [5]. Patients with unsolved problems and unmet needs experience poor health status and quality of life [6]. Assessing patients’ care needs in a given setting therefore is important for developing tailored palliative care services to overcome their problems and meet their needs. Healthcare services that are inconsistent with their care needs would increase healthcare cost and result in negative effects such as increasing patient’s anxiety and deteriorating quality of life [7].

Providing tailored palliative care services requires systematic and comprehensive assessment first, and such an assessment could be supported better by a valid and specific instrument [8]. Several instruments have been developed and used in advanced cancer patients, and they have been critically evaluated in a recent systematic review conducted by our group [5]; not all the assessed instruments reported adequate evidence with regards to their psychometric properties [5]. Multidimensional instruments with well-documented psychometric properties were scant and the commonly used scales in current practice and research were the Supportive Care Needs Survey (SCNS), Problems and Needs in Palliative Care questionnaire (PNPC), and the Needs Assessment of Advanced Cancer Patients (NA-ACP) [2]. The SCNS is a generic tool rather than a specific one for patients at advanced stage. The two other instruments (PNPC and NA-ACP) were particularly designed for advanced cancer patients and cover the majority of the palliative care needs of advanced cancer patients [9, 10]. However, the NA-ACP has 132 items, which might overburden patients and contribute to missing data [11]. Besides, patients might not want professional care support for each of the problems they experience [12]. The PNPC questionnaire was designed with considering this issue, and it assesses patients’ problems and to which extent they want care support to address their problems (needs-for-care) separately [10].

The PNPC questionnaire was first developed in 2004 through a series of rigorous procedures including in-depth interviews with patients, their life companions and health professionals, critical literature review, expert panel discussion, and repeated adjustment [10]. The original language of the PNPC questionnaire was Dutch and it has been translated into English [10]. The original version consisted of 90 items, thus patients still needed a long time to complete the entire questionnaire. To improve its feasibility and utility, the PNPC-shorter version (PNPC-sv) with 33 items was subsequently developed in 2007 [13]. Its psychometric properties have been examined, with adequate validity, reliability and feasibility [13]. The PNPC-sv, as a simple and user-friendly instrument, has been translated and utilized in both research and clinical practice in some countries [14, 15]. Due to the absence of such an instrument in China, the aims of this study were to translate the PNPC-sv questionnaire into Mandarin Chinese and determine its reliability, validity and feasibility in Chinese patients with advanced cancer.

## Methods

### Study design

This was a validation study using a forward- and backward- translation procedure, a panel of experts and a cross-sectional study design, from October 2017 to April 2018. A convenience sampling approach was used for subject recruitment. Ethical approvals were granted by the Human Subjects Ethics Sub-Committee at The Hong Kong Polytechnic University and the study hospitals. Written informed consent was required from each study participant.

### Translation of the PNPC-sv

The original English version was translated into Mandarin Chinese following a forward- and backward-translation procedure [16]. Four independent translators (two each for forward and backward translation) were included with the following inclusion criteria [16]: (1) were bilingual, including English and Mandarin Chinese; and (2) had different backgrounds, were knowledgeable about the terminology used in health science, and were familiar with colloquial phrases, idiomatic expressions, and health care slang and jargon in Mandarin Chinese.

#### Forward translation

The original English version of the PNPC-sv was translated into Mandarin Chinese by two translators separately. The first author and a nursing academic (who was bilingual in English and Mandarin Chinese, had a PhD degree in nursing and has accumulated extensive research experience in cancer and palliative care) compared the two translated Chinese versions from ambiguities and discrepancies of the words and sentences. Different translations were identified for five items (item 14, 19, 20, 27, 28). Discussions among the researcher, nursing academic and the two translators were conducted to reach an agreement.

#### Backward translation

The preliminary translated Chinese version of PNPC-sv was translated back into English by two other independent translators who were blind to the original English version. The equivalence of the original and back-translated English versions were assessed and compared by a panel, consisting of the researcher, all four translators and the nursing academic. Different opinions were raised regarding items 20, 27 and 28 (item 20—‘Finding others not receptive to talking about the disease’; item 27—‘Difficulties to be engaged usefully’; and item 28—‘Difficulties to be avail for others’). Discussions were conducted among the panel and further revisions were made to reach agreement.

### Readability and clarity test of the translated version

As recommended by Koller et al., [17], 10 adult (> 18 years old) Chinese advanced cancer patients (stage III or stage IV) with different background (e.g., education level, age, and occupation) were included using a purposive sampling approach. After they completed the entire questionnaire, participants were asked six questions regarding the readability and clarity of the PNPC-sv to determine if the items in the questionnaire were easy to read and understand and if any of the items have particular ambiguous, discrepant and sensitive expressions. Those six questions were designed based on previous studies [18, 19] and group discussions among the researchers: Q1) “Is the instrument useful to record your problems and needs that you experienced during your cancer trajectory? [0-10 numerical rating scale [NRS] from 0 (totally useless) to 10 (totally useful)]”; Q2) “Is the instrument easy for you to complete? [0-10 NRS scale from 0 (extremely difficult) to 10 (extremely easy)]”; Q3) “Are there any difficulties in understanding any of the items? (yes /no, no=0, yes=1)? If yes, please specify.”; Q4) “Are there any sensitive items or words that make you do not want to fill out the instrument? (yes /no, no=0, yes=1)? If yes, please specify.”; Q5) “How long it takes you to complete the instrument (minutes)?”; and Q6) Do you have any other comments and recommendations? Please specify".

Participants reported that the PNPC-sv Mandarin Chinese version can comprehensively assess their existing problems, with the score of Q1 rating from 7 to 10 (mean: 8.5). The PNPC-sv was also regarded as a scale easy to understand (Q2 mean: 8.5), and the average time for completing the questionnaire was 11.4 min. No patient complained about sensitive and/or abstract words or items. The completion rate was high, without any missing data in any item. This translated version was confirmed with satisfactory readability and clarity and was further used in the next study phase to examine its psychometric properties.

### Sample and sample size calculation

Eligible patients were recruited from three tertiary hospitals in China based on the following inclusion criteria: (1) a confirmed diagnosis of cancer at advanced stage (stage III or stage IV), (2) aged above 18 years, (3) able to communicate in Mandarin Chinese, (4) willing to take part in this study and sign the informed consent, and (5) emotionally, cognitively and physically capable of study participation.

Cengiz, et al. [20] proposed that the sample size for estimating the reliability of an instrument should be 5 to 10 times larger than the total items of the scale. The PNPC-sv has 33 items, and the sample size therefore should be at least 165 patients. Hobart et al. [21] suggested that 20 and 80 subjects were the minimal sample size for the reliability and validity estimation, respectively. Considering the above recommendations, 165 was used as the estimated sample size of this study. By considering additional 8% of missing data, the sample size was finally determined as 178.

### Data collection procedure

The content validity of the translated version of the PNPC-sv was evaluated through a panel of six experts who were specialized in cancer care and/or palliative care. Of which, three experts had more than 15 years of clinical or research experience, with the title of professor or associate professor in universities or tertiary hospitals. The other three were lecturers or senior lecturers with more than 5 years of experience in cancer-related research. Four experts had a doctoral degree and two had a master’s degree. The panel used a four-point Likert scale (“4 = very relevant”, “3 = quite relevant”, “2 = somewhat relevant” and “1 = not relevant”) to assess the cultural relevance and translation equivalence of each item. Oncologists or oncology nurses helped to screen and identify the eligibility of the patients at the study hospitals. Detailed information of the study objectives and procedures were elaborated by the researcher before inviting them to participate in this study. Patients who agreed with study participation were asked to sign a written consent. Each patient was then asked to complete a demographic questionnaire, the translated Chinese PNPC-sv questionnaire and the EORTC QLQ- C30. Participants completed all the questionnaires anonymously and they returned the questionnaires to the researchers immediately after completion. For any missing data or scribbled answer, the participants were asked for clarification. The PNPC-sv was self-administered, and the researchers provided assistance to patients who were unable to fill in the questionnaire on their own by reading the items as they were in the scale and not providing any further clarification.

### Study questionnaires

#### Demographic questionnaire

A demographic questionnaire was specifically designed for this validation study. The items included age, gender, educational background, income level, place of residence, religion and marital status, and illness-related information including diagnosis, cancer stage and relevant treatments, etc.

#### Problems and needs in palliative care-short version (PNPC-sv)

The PNPC-sv has 33 items and covers eight domains of problems and palliative care needs of advanced cancer patients including daily activities (3 items), physical (9 items), autonomy (4 items), social (5 items), psychological (5 items), spiritual (4 items), financial (2 items) and informational (1 item) issues [13]. The PNPC-sv consists of the *problem* part and the *need-for-care* part [13]. In each item, the patients were asked two questions [13]: (1) “Do you experience the item to be a problem?”, which belongs to the *problem* part with the answer of “*yes”, “somewhat”,* and *“no”*; and (2) “Do you need (extra) professional attention for the item?”, which belongs to the *need-for-care* part with the answer of “*yes, more*”, “*as much as now*” and “*no*”. In terms of the PNPC-sv scoring system for the psychometric assessment purpose, the scoring method of the original questionnaire [13] and the recommendations from the researcher who developed the PNPC-sv were adapted (“yes” = 2, “somewhat”/ “as much as now” =1, and “no” = 0). Higher scores indicate more problems and stronger care needs. The psychometric properties of the *problem* part and the *need-for-care* part were determined separately.

#### European Organisation for Research and Treatment of Cancer (EORTC) quality-of-life questionnaire Core 30 (EORTC QLQ-C30)

As with the original version of PNPC-sv, the QLQ-C30 was used to test the concurrent validity of the PNPC-sv Mandarin Chinese version. This scale is a self-administered QoL scale which was specifically designed for cancer patients [22]. It consists of 30 items, with five scales assessing functional status (physical, role, emotional, cognitive, and social), three scales assessing symptoms (pain, fatigue, and nausea and vomiting), one scale measuring global health status/QoL, and some single items measuring other symptoms which are frequently reported by cancer patients, and one item regarding financial difficulties [22]. Higher scores for each subscale indicate poorer QoL. While for the global health status /QoL scale, higher score represents better QoL. Satisfactory psychometric properties of the QLQ-C30 have been reported in Chinese cancer patients [23].

### Psychometric properties

#### Validity

Content validity, face validity, concurrent validity and construct validity were measured. A panel of six experts identified the content validity through a four-point Likert scale. Face validity was examined by asking patients and experts several questions regarding the feasibility, usability and clarity of the PNPC-sv. Concurrent validity is “how well a test correlates with another test that has already had its validity estimated” [24] (p. 53), which was examined by exploring the relationships between the PNPC-sv and the QLQ-C30. Subscales in the PNPC-sv that do not have corresponding dimensions in QLQ-C30 were not included in the concurrent validity test [13]. The total scores and sub-scores of the PNPC-sv and the QLQ-C30 were hypothesized to be significantly correlated with each other. For construct validity, confirmatory factor analysis was performed to evaluate the fitness of original model of the PNPC-sv to the present data. Besides, the construct validity was also evaluated using contrasted group analysis, which is an approach used for identifying differences between known groups to demonstrate different traits on a construct of measurement [25]. Based on previous studies [5, 26], differences in the total scores and sub-scores of the PNPC-sv were compared between patient subgroups with different gender, age, marital status, educational level, living place, and cancer stage. We hypothesized that female patients would have higher scores in the psychological, physical and ADL subscales; single patients would demonstrate higher scores in the psychological subscale; scores of financial problems and needs would be higher among patients with lower education and those living in countryside; elderly patients would report higher scores in terms of ADL and physical subscales, but lower financial scores; and the scores of physical and psychological subscale would be higher among stage IV cancer patients [5, 26].

#### Reliability and acceptability

Internal consistency reliability of the PNPC-sv Mandarin Chinese version was examined using Cronbach’s alpha [27]. Item-to-total correlations were measured to test how well each item score correlates with the overall PNPC-sv score [28]. Test-retest reliability was not measured given that problems and palliative care needs of advanced cancer patients are not stable as they usually experience rapid progression or deterioration [1]. Completion rate and the six questions (has mentioned before) were used to determine its acceptability and feasibility.

### Data analysis

Data analysis was conducted using the IBM SPSS 22.0 and the IBM SPSS Amos 24.0. All statistical tests were two-tailed and the significance level was set as *P* < 0.05. Descriptive statistics were used to present the demographic characteristics of the patients. The content validity index (CVI) was used to measure content validity of the scale. CVI for each PNPC-sv item was examined by the proportion of items which were rated as “very relevant” or “quite relevant” [29–31] by the expert panel. The item was regarded as content valid when at least five out of six experts rating it as “very relevant” or “quite relevant” [31]. The average CVI across items was used to present the content validity of the entire PNPC-sv scale, and a CVI of 0.83 or above was viewed as a satisfactory agreement level [29, 31]. Structural Equation Modelling was used to evaluate the relationships between structural paths and factors. The goodness-of-fit indicators including Chi-square (x^2^) divided by degrees of freedom (x^2^/df), Root-Mean-Square Error of Approximation (RMSEA), the Comparative Fit Index (CFI), the Tucker-Lewis index (TLI), and the Root Mean square Residual (RMR) were employed to assess the fit of the original model to this sample data [32]. The criteria for good fit were 1.0 < x^2^/df < 3.0, RMSEA≤0.08, CFI≧0.90, TLI≧0.90, and RMR ≦0.05 [33]. If the model does not fit the data adequately, items with factor loading of 0.4 or below are considered to be removed [34], but whether it is deleted or not would be finally determined based on both the *statistical* and *judgmental* criteria for scale-purification [35] (details is elaborated in the discussion section). The normality of each independent variable (demographic characteristics) was explored by using the Kolmogorov-Smirnov tests. A Mann-Whitney U test was finally utilized for the contrasted group analysis due to the non-normal distribution of the variables. For concurrent validity, Spearman’s correlations were used to explore the relationships between the PNPC-sv and the QLQ-C30. A correlation coefficient of 0.40 or above was regarded as substantial for conceptually related scales [36, 37]. Reliability was tested using Cronbach’s alpha and item-to-total correlations. Alpha values and values of item-to-total correlations were regarded as acceptable when they reached 0.65 and 0.40 or above, respectively [38].

## Results

### A: psychometric properties assessed by the expert panel

#### Content validity and face validity

A panel of six experts were invited and two rounds of content validity assessment were performed. In round one, some suggestions and comments were obtained for item 10, 17 and 29. For example, three experts suggested changing the “sexual dysfunction” (item 10) to “… affecting sexual life”, which could make this expression less sensitive within the conservative Chinese culture [11]. With considering those suggestions and comments, item 10 was revised. In the second-round, all six experts agreed that the PNPC-sv is specifically designed for measuring problems and palliative care needs of advanced cancer patients and all items are culturally appropriate. A CVI of 1.0 was achieved at both the item-level and the scale-level.

Patients further reported that the PNPC-sv is a useful instrument to assess their problems and needs and the mean score was 7.99 (SD = 1.48). Almost all of the participants reported that the items were not particularly sensitive and easy to understand. Only one patient reported the item 16 (“experiencing loss of control over one’s life”) was a little difficult to understand, and item 10 (“affecting sexual life”) and item 29 (“difficulties concerning the meaning of death”) were reported somewhat sensitive by three and one patient, respectively.

### B: psychometric properties assessed via the patients

#### Demographic and clinical characteristics of the patients

One hundred and seventy-eight patients were included and 174 completed all the questionnaires. 96.6% of the patients were recruited from inpatient settings. More than 60% of the patients were male and younger than 60 years old. 75.9% had a middle school education or below, and a majority of the patients were married (94.8%) and employed (80.5%). The common diagnoses of patients were lung cancer, nasopharynx cancer and colorectal cancer, and nearly 60% of the patients were at stage IV (Table 1).

**Table 1:** Demographic and clinical characteristics of the study sample (*N* = 174)

Demographic and clinical characteristics		*N* (%)
Age (year)	< 60	109 (62.6%)
	≥60	65 (37.4%)
Gender	Female	69 (39.7%)
	Male	105 (60.3%)
In/outpatient	Inpatient	168 (96.6%)
	Outpatient	6 (3.4%)
Educational level	Middle school education or below	132 (75.9%)
	High school education or above	42 (24.1%)
Marital status	Single	9 (5.2%)
	Married	165 (94.8%)
Employment status	Technical staff	24 (13.8%)
	Manual worker	57 (32.8%)
	Housewife	10 (5.7%)
	Clerical/admin	17 (9.8%)
	Self-employed	32 (18.4%)
	Unemployment	8 (4.6%)
	Retired	26 (14.9%)
Religion	Non/Not Indicated	145 (83.3%)
	Buddhism	26 (14.9%)
	Taoism	3 (1.8%)
Location of living place	Countryside	80 (46.0%)
	City	94 (54.0%)
Living status	Living alone	3 (1.7%)
	Living with family	171 (98.3%)
Types of cancer	Lung cancer	54 (31.0%)
	Nasopharynx cancer	30 (17.2%)
	Colorectal cancer	29 (16.7%)
	Gynecological cancer	32 (18.4%)
	Liver cancer	5 (2.9%)
	Breast cancer	4 (2.3%)
	Esophageal cancer	3 (1.7%)
	Oral cancer	6 (3.4%)
	Others	11 (6.4%)
Stage of cancer	III	70 (40.2%)
	IV	104 (59.8%)

#### Acceptability and descriptive analysis of the scale

Acceptability of the PNPC-sv was satisfactory with the completion rate of 97.6%. The majority of the patients reported the PNPC-sv is easy to understand. The average time to complete the questionnaire was 11 min. Percentages of each PNPC-sv item reported to be either a problem or *somewhat* a problem by the patients ranged from 7.5 to 83.9%, with financial problems were the most prominent issue (69.5 to 83.9%). For the indicated problems, 10.3% of the patients had the need for professional attention and support. (Table 2).

**Table 2:** Cronbach’s alpha reliability: Total scale and subscale

PNPC-sv dimension	No. of items	PNPC *Problem* part		PNPC *Need-for-care* part	
		Range in percentage of “somewhat” and “yes” (%)	Cronbach’s alpha	Range in percentage of “as much as now” and “yes”	Cronbach’s alpha
ADL	3	30.5–42.0	0.75	27–29.3	0.81
Physical	9	20.1–54.0	0.61	13.2–54.3	0.72
Autonomy	4	25.1–52.3	0.79	27–45.4	0.84
Social	5	7.5–23.6	0.75	10.3–20.1	0.79
Psychological	5	30.5–49.4	0.78	23–42.5	0.85
Spiritual	4	19.5–38.5	0.68	19.5–27.6	0.80
Financial	2	69.5–83.9	0.58	63.2–82.8	0.69
Information	1	42.5	NA	43.1	NA
Total scale			0.88		0.91

Note: NA=not applicable

#### Reliability

Cronbach’s alpha coefficient for the total scale of *problem* part and *need-for-care* part was 0.88 and 0.91, respectively. Cronbach’s alpha coefficients for all the subscales within the *problem* part ranged from 0.58–0.79, while they were 0.69–0.85 for the subscales within the *need-for-care* part (Table 2). The majority of the item-to-total correlations were above 0.40.

#### Construct validity

The goodness of fit indexes for the *problem* part were x^2^ = 700.8, x^2^/df = 1.58, RMSEA = 0.06, CFI = 0.83, TLI = 0.81, and RMR = 0.04. For the *need-for-care* part, the corresponding indexes were x^2^ = 907.354, x^2^/df = 2.05, RMSEA = 0.07, CFI = 0.81, TLI = 0.79, and RMR = 0.03. The CFI and TLI in both the *problem* part and *need-for-care* part were slightly below the cut-off values of 0.90. The factor loading ranged from 0.12 to 0.79 for *problem* part and from 0.23 to 0.87 for *need-for-care* part. The items with factor loading less than 0.4 were all in the physical factor including item 8- ‘Itch’, item 9- ‘Sexual dysfunction’, item 10- ‘Prickling or numb sensation’ and item 11- ‘(Nightly) Sweating or hot flushes’. Considering that all these four symptoms were not uncommon theoretically and clinically in advanced cancer patients, the four items were not deleted after a group discussion with clinicians and researchers in cancer care to maintain its clinical value.

#### Contrasted groups validity

Female participants reported higher scores regarding global and some sub-scores including ADL, psychological, and spiritual domains (*P* < 0.05) for both the *problem* part and *needs-for-care part* (Table 3**)**. Higher scores were presented in older patients regarding ADL, physical, social problems and the global score of the *problem* part (*P* < 0.05). Younger patients demonstrated more financial problems (*P* < 0.05). Similar results were detected for the *needs-for-care* part, with older patients having higher needs scores for ADL, physical, and social support (*p* < 0.05), and older patients reported lower score for financial needs (*P* < 0.05) (Table 4**)**. Patients who were living in countryside had higher scores for the financial needs (*P* < 0.001). Except for psychological needs (*P* < 0.05), no significant differences were detected in the marital status of patients. Single patients had lower scores of psychological needs (*P* = 0.045). In terms of the educational level, patients with middle school education or below reported higher scores of financial problems (*P* < 0.01) and financial needs (*P* < 0.001). Stage IV cancer patients had higher scores regarding the autonomic and social problems (*P* < 0.05) as well as higher global score of the *problem* part (*P* < 0.05) than patients with stage III cancer. Similar trend was detected in the *needs- for-care* part.

**Table 3:** Difference in total and sub-scores of PNPC-sv between female and male subjects

Dimensions	Female		Male		Z value	*P* value
	*n*	Mean Rank	*n*	Mean Rank		
Problem part						
ADL	69	99.63	105	79.53	−2.685	0.007^a^
Physical	69	92.66	105	84.11	−1.100	0.271
Autonomy	69	91.93	105	84.59	−0.962	0.336
Social	69	87.04	105	87.80	−0.113	0.910
Psychological	69	101.54	105	78.28	−3.017	0.003^a^
Spiritual	69	98.29	105	80.41	−2.400	0.016^a^
Financial	69	87.67	105	87.39	− 0.038	0.970
Information	69	94.79	105	82.71	−1.746	0.081
Global Problem Score	69	98.28	105	80.27	−2.338	0.019^a^
Need-for-care part						
ADL	69	99.26	105	79.77	−2.728	0.006^a^
Physical	69	91.50	105	84.87	−0.857	0.392
Autonomy	69	93.17	105	83.78	−1.265	0.206
Social	69	84.64	105	89.38	−0.731	0.465
Psychological	69	100.11	105	79.21	−2.776	0.006^a^
Spiritual	69	97.03	105	81.24	−2.230	0.026^a^
Financial	69	88.44	105	86.88	−0.211	0.833
Information	69	92.75	105	84.05	−1.255	0.210
Global Problem Score	69	97.28	105	81.07	−2.079	0.038^a^

Note: a=Statistic reached a level of statistical significance

**Table 4:** Difference in total and sub-scores of PNPC-sv between different age groups

Dimensions	<60ys		≥60ys		Z value	*P* value
	*n*	Mean Rank	*n*	Mean Rank		
Problem part						
ADL	109	81.14	65	98.17	−2.250	0.024^a^
Physical	109	80.78	65	98.77	−2.288	0.022^a^
Autonomy	109	83.53	65	94.16	−1.379	0.168
Social	109	81.06	65	98.30	−2.507	0.012^a^
Psychological	109	84.99	65	91.71	−.862	0.389
Spiritual	109	83.89	65	93.55	−1.281	0.200
Financial	109	94.87	65	75.15	−2.699	0.007^a^
Information	109	83.94	65	93.47	−1.362	0.173
Global Score	109	81.55	65	97.48	−2.020	0.043^a^
Need-for-care part						
ADL	109	81.83	65	97.00	−2.099	0.036^a^
Physical	109	79.51	65	100.90	−2.734	0.006^a^
Autonomy	109	84.68	65	92.23	−1.006	0.314
Social	109	80.79	65	98.75	−2.743	0.006^a^
Psychological	109	83.94	65	93.48	−1.253	0.210
Spiritual	109	84.82	65	92.00	−1.003	0.316
Financial	109	93.45	65	77.52	−2.130	0.033^a^
Information	109	83.72	65	93.85	−1.444	0.149
Global Score	109	81.77	65	97.11	−1.945	0.052

Note: a=Statistic reached a level of statistical significance

#### Concurrent validity

Significant positive correlations were found between PNPC-sv and QLQ-C30 in terms of the majority of the subscale scores, with the correlation coefficients ranging from 0.19 to 0.56 in the *problem* part and from 0.24 to 0.60 in the *need-for-care* part. Significant negative correlations were identified between the total score of PNPC-sv and the score of global health status of QLQ-C30, with the correlation coefficient of − 0.48 and − 0.42 for the *problem* part and *need-for-care* part, respectively. Correlation identified between the *problem* part of PNPC-sv and QLQ-C30 was better than that between the *need-for-care* part of PNPC-sv and QLQ-C30 for the majority of the subscales (Table 5).

**Table 5:** Correlations between the PNPC-sv and the EORCTQOL-C30

PNPC-sv dimension	QOL-C30 dimensions expected to correlate	PNPC *problem* part	PNPC *need-for-care* part
		Spearman’s Correlation	Spearman’s Correlation
ADL	Physical functioning	0.563^a^	0.597^a^
Physical	Fatigue	0.509^a^	0.588^a^
	Nausea and vomiting	0.200^a^	0.129
	Pain	0.581^a^	0.519^a^
	Dyspnoea	0.509^a^	0.337^a^
	Insomnia	0.437^a^	0.358^a^
	Appetite loss	0.196^a^	0.250^a^
Financial	Financial difficulties	0.363^a^	0.477^a^
Social	Social functioning	0.188^b^	0.243^a^
Psychological	Emotional functioning	0.527^a^	0.499^a^
Global scores	Global health status	−0.484^a^	−0.419^a^

**Note:** This table shows Spearman’s rho correlations of sum scores of proposed PNPC-sv dimensions with corresponding dimensions of the EORTC QLQ-C30. ^a^The correlations are significant at 0.01. ^b^Significant at 0.05

## Discussion

The PNPC-sv is currently the only scale developed to evaluate both the problems of advanced cancer patients and to which extent they need for care support to address their problems (palliative care needs). The PNPC-sv was initially developed in Dutch [10], and it was subsequently translated into English [13] and Indonesian [15]. The psychometric properties of the Indonesian version were not reported [15]. This paper presents the first validation study of the PNPC-sv Mandarin Chinese version in advanced cancer patients. Conceptual and cultural equivalence between the original and the Mandarin Chinese version of the PNPC-sv were well maintained through the approach of forward- and backward- translation, which enables the Mandarin Chinese version of the PNPC-sv to be culturally relevant to Chinese advanced cancer patients [39]. Excellent content validity was identified with the CVI value being higher than 0.83 [29, 31]. Face validity was documented, as patients reported that the PNPC-sv questionnaire can comprehensively cover and assess their existing problems and palliative care needs. Usability and clarity of this tool were well supported by the responses of both the panel experts and the patients. Given patients completing the questionnaire within a relatively short time and the good completion rate, the PNPC-sv was proved to be a convenient and user-friendly tool. Such a convenient instrument will produce less burden on patients and minimizes the risk of missing data.

Concurrent validity of the PNPC-sv was adequate with moderate or strong correlations identified in majority of the subscales, which was similar to the original version [13]. The significant negative associations between the total scores of the PNPC-sv and global health status of the QLQ-C30 supported that patients who had more problems and care needs experienced poorer health status and QoL [6]. Statistically significant correlations were also observed between the majority of the subscales of PNPC-sv (*problem* and *need-for-care* part) and QLQ-C30, confirming that the PNPC-sv and the QLQ-C30 are conceptually related. As expected, the correlations of QLQ-C30 were statistically stronger regarding the *problem* part than the *need-for-care* part and weak correlations were identified for a few subscales. Such findings were similar to the psychometric assessment results of the original version [13]. A possible explanation might be the difference in the focus of the PNPC-sv and the QLQ-C30. The QLQ-C30 scale mainly assesses patients’ quality of life through capturing the problems they experienced, while patients’ needs for professional care are not its focus. Compared with the psychometric assessment study of the original version of the PNPC-sv, significant correlations between the PNPC-sv and the QLQ-C30 were observed in more subscales in this study, which may be partially attributed to a larger sample size in the current study.

Factor analysis has been regarded as one of the commonly utilised methods in psychological measures development and evaluation. In this study, the value of CFI and TLI were slightly lower than the recommended cut-off points (0.90), which might indicate that the original model did not well fit this sample data adequate. However, the CFI and TLI value were close to the threshold of 0.90. Meanwhile, according to the critical value of 0.80 proposed by Kline [40], the results may indicate that the overall fit of the instrument model was basically acceptable. Some also argued that ‘if the vast majority of the indexes indicate a good fit, then there is probably a good fit’ (p. 327) [32]. In this study, three out of five fit indexes meet the critical criteria, which might, to some extent, indicate a potentially acceptable fit. Removing items with low or complex factor loadings is a commonly used approach when the hypothesized model does not fit the data adequate [41]. However, researchers should “ensure that *judgmental* and *statistical* criteria are combined before making a scale purification decision” (p. 325) [35]. The application of *judgmental* criteria mainly relies on theoretical and practical knowledge of the domain experts [35]. *Judgmental* assessment ensures that “a scale covers the entirety of all relevant aspects that need to be measured” (p. 325) [35]. Thus, whether the items with low factor loading can be definitely deleted should be determined based on not only the statistical results but also researchers’ professional and practical knowledge and concerns. In this study, the PNPC-sv was a clinical practice-focused instrument which aims at examining problems and to which extent the available care can address the problems in general advanced cancer patients. Keeping these items in the scale therefore would maintain the clinical value of this instrument and help clinicians comprehensively identify patients’ physical problems and needs. All the physical symptoms mentioned in the items were not uncommon in advanced cancer patients, and they were “regarded as relevant from a theoretical perspective” (p. 216) [42]. According to a general rule of thumb, the sufficient sample size for confirmatory factor analysis should be 300–500 subjects [16, 43], while the sample size in this study was significantly fewer than the recommended sample size. In such a relatively small sample size, a mixed sample with more than 10 types of cancer were included, which might be a possible reason to contribute to the low factor loadings as those symptoms were particularly related to specific cancer types or cancer treatments. Thus, the currently study results can only be interpreted as preliminary given the mixed study sample with various types of cancer and a relatively small sample size.

Construct validity of the PNPC-sv was well demonstrated given contrasted groups analysis clearly indicated that patients with different gender, age, living place and cancer stage presented different problems and care needs in some specific PNPC-sv domains. Female patients reported more ADL, psychological and spiritual problems and needs, and the results were consistent with previous studies [44, 45]. Living in rural or urban areas has been deemed as an influencing factor for palliative care needs of advanced cancer patients [26], which was also verified in this study. The results of elderly patients having more physical issues and fewer financial issues were consistent with some previous studies [44, 46], although opposite results were identified in some other studies, with elderly patients reporting fewer physical issues [47, 48]. Patients with stage IV cancer generally showed more problems and higher needs, which was in line with only one previous study [49]. It might be because the predictive value of age and cancer stage on problems and care needs are not as strong as the gender factor, and this study adopted non-parametric tests which are less powerful than parametric tests. The factors of age and cancer stage are worthy of further exploration. Different from previous studies, statistical differences were detected in only one subscale of the PNPC-sv among patients of different educational level and marital status, and the considerably uneven sample size between groups may partially contribute to this. Reliability was adequate and it was similar to the original version, which indicates that the Mandarin Chinese PNPC-sv is internally reliable. Acceptable internal consistency indicated that items of each domain of the PNPC-sv measure the same construct and conceptually fit together [50].

There were some limitations of this study. Although the patients of this study were recruited from three study sites, the convenience sampling method used for subject recruitment may limit the generalizability of the study findings. A mixed sample with various types of cancer diagnosis in this study contributed to significantly heterogeneity of the study participants, and results from the factor analysis should be prudently interpreted. Future research is needed to further examine the psychometric properties of the PNPV-sv, particular its internal structure, in new and larger patient samples.

## Conclusion

The Mandarin Chinese version of the PNPC-sv is a valid, reliable and user-friendly instrument for measuring problems and palliative care needs of patients with advanced cancer. The PNPC-sv can facilitate the clinicians and researchers to better identify specific palliative care needs of advanced cancer patients within the Chinese contexts, and subsequently provide evidence to develop tailored palliative care services. However, instrument validation is an ongoing process and future psychometric studies in larger samples using stratified random sampling are needed to further examine the psychometric properties of the PNPV-sv and improve its generalizability.

## Data Availability

The datasets used and/or analysed in this study are available from the corresponding author for a reasonable request.

## Abbreviations

ADL: Activities of Daily Life
CFI: The Comparative Fit Index
CVI: Content Validity Index
EORTC QLQ-C30: EORTC Quality-of-Life Questionnaire-Core 30
NA-ACP: Needs Assessment of Advanced Cancer Patients
PNPC: Problems and Needs in Palliative Care questionnaire
PNPC-sv: Problems and Needs in Palliative Care questionnaire-short version
RMR: Root Mean square Residual
RMSEA: Root-Mean-Square Error of Approximation
SCNS: Supportive Care Needs Survey
TLI: The Tucker-Lewis index
x^2^: Chi-square
x^2^/df: Chi-square divided by degrees of freedom

## References

[1] Waller A, Girgis A, Johnson C (2012). Improving outcomes for people with progressive cancer: interrupted time series trial of a needs assessment intervention. J Pain Symptom Manag.

[2] Sanson-Fisher R, Girgis A, Boyes A (2000). The unmet supportive care needs of patients with cancer. Cancer.

[3] Chu FY. Predicting quality of life in Taiwanese women with breast cancer and the role of complementary and alternative medicine: a mixed-method study: Griffith University, Queensland, Australia. PhD thesis; 2009.

[4] World Health Organization. WHO definition of palliative care. http://www.who.int/cancer/palliative/definition/en/. Accessed 25 Mar 2019.

[5] Wang T, Molassiotis A, Chung BPM (2018). Unmet care needs of advanced cancer patients and their informal caregivers: a systematic review. BMC Palliat Care.

[6] Cheng KKF, Wong WH, Koh C (2016). Unmet needs mediate the relationship between symptoms and quality of life in breast cancer survivors. Support Care Cancer.

[7] Wen KY, Gustafson DH (2004). Needs assessment for cancer patients and their families. Health Qual Life Outcomes.

[8] Wang T, Molassiotis A, Chung B (2018). Current research status of palliative Care in Mainland China. J Palliat Care.

[9] Rainbird KJ, Perkins JJ, Sanson-Fisher RW (2005). The needs assessment for advanced Cancer patients (NA-ACP): a measure of the perceived needs of patients with advanced, incurable cancer. A study of validity, reliability and acceptability. Psycho-Oncology.

[10] Osse BH, Vernooij MJ, Schadé E (2004). Towards a new clinical tool for needs assessment in the palliative care of cancer patients: the PNPC instrument. J Pain Symptom Manag.

[11] Tan JY. Effects of auricular acupressure on chemotherapy-induced nausea and vomiting in breast cancer patients: a preliminary randomized controlled trial: The Hong Kong Polytechnic University. PhD Thesis; 2017. Retrieved from: http://ira.lib.polyu.edu.hk/handle/10397/72590 10.1186/s12906-022-03543-y PMC8953362 35331208

[12] Steinert Y, Rosenberg E (1987). Psychosocial problems: what do patients want? What do physicians want to provide?. Fam Med.

[13] Osse BHP, Vernooijdassen MJFJ, Schadé E (2007). A practical instrument to explore patients’ needs in palliative care: the problems and needs in palliative care questionnaire - short version. Palliat Med.

[14] Khan L, Chiang A, Barnes E (2012). Needs assessment of patients and their caregivers at the rapid response radiotherapy program. J Pain Manag.

[15] Effendy C, Vissers K, Osse BHP (2015). Comparison of problems and unmet needs of patients with advanced Cancer in a European country and an Asian country. Pain Pract.

[16] Sousa VD, Rojjanasrirat W (2011). Translation, adaptation and validation of instruments or scales for use in cross-cultural health care research: a clear and user-friendly guideline. J Eval Clin Pract.

[17] Koller M, Aaronson NK, Blazeby J (2007). Translation procedures for standardised quality of life questionnaires: the European Organisation for Research and Treatment of Cancer (EORTC) approach. Eur J Cancer.

[18] Kakehi Y, Kamoto T, Ogawa O (2002). Development of Japanese version of the UCLA prostate Cancer index: a pilot validation study. Int J Clin Oncol.

[19] Tan JY, LKP S, Molassiotis A (2016). Psychometric assessment of the Chinese version of the MASCC Antiemesis tool (MAT) for measuring chemotherapy-induced nausea and vomiting. Support Care Cancer.

[20] Cengiz E, Ozsari H, Akyuz I (2015). Private hospital choices of infertile patients that received IVF treatment: a pilot study. Eur Sci J.

[21] Hobart JC, Cano SJ, Warner TT (2012). What sample sizes for reliability and validity studies in neurology?. J Neurol.

[22] Aaronson NK, Ahmedzai S, Bergman B (1993). The European Organization for Research and Treatment of Cancer QLQ-C30: a quality-of-life instrument for use in international clinical trials in oncology. J Natl Cancer Inst.

[23] Wan C, Meng Q, Yang Z, Tu X, Feng C, Tang X (2008). Validation of the simplified chinese version of eortc qlq-c30 from the measurements of five types of inpatients with cancer. Ann Oncol J Eur Soc Med Oncol.

[24] Newman I, Newman C (1994). Conceptual Statistics for Beginners.

[25] Terwee CB, Bot SD, de Boer MR, van der Windt DA, Knol DL, Dekker J, Bouter LM, de Vet HC (2007). Quality criteria were proposed for measurement properties of health status questionnaires. J Clin Epidemiol.

[26] Fitch M. Providing supportive care for individuals living with cancer. Ont Cancer Treat Res Found. 1994; Toronto.

[27] Frost MH, Reeve BB, Liepa AM, Stauffer JW, Hays RD (2007). what is sufficient evidence for the reliability and validity of patient-reported outcome measures?. Value Health.

[28] Bohrnstedt GW (1969). A quick method for determining the reliability and validity of multiple-item scales. Am Sociol Rev.

[29] Rubio DM, Berg-Weger M, Tebb SS (2003). Objectifying content validity: conducting a content validity study in social work research. Soc Work Res.

[30] Waltz CF, Bausell BR. Nursing research: design statistics and computer analysis. Philadelphia: F.A. Davis Co; 1981.

[31] Lynn MR (1986). Determination and quantification of content validity. Nurs Res.

[32] Schreiber JB, Nora A, Stage FK, Barlow EA, King J (2006). Reporting structural equation modeling and confirmatory factor analysis results: a review. J Educ Res.

[33] Arpaci I, Baloğlu M (2016). The impact of cultural collectivism on knowledge sharing among information technology majoring undergraduates. Comput Hum Behav.

[34] Malakouti SK, Fatollahi P, Mirabzadeh A, Salavati M, Zandi T (2006). Reliability, validity and factor structure of the GDS-15 in Iranian elderly. Int J Geriatr Psychiatry.

[35] Wieland A, Durach CF, Kembro J, Treiblmaier H (2017). Statistical and judgmental criteria for scale purification. Supply Chain Management. Int J.

[36] Kaasa S, Bjordal K, Aaronson N (1995). The EORTC core quality of life questionnaire (QLQ-C30): validity and reliability when analysed with patients treated with palliative radiotherapy. Eur J Cancer.

[37] Lim LL, Seubsman SA, Sleigh A (2008). Thai SF-36 health survey: tests of data quality, scaling assumptions, reliability and validity in healthy men and women. Health Qual Life Outcomes.

[38] DeVellis RF (1991). Scale development: theory and applications.

[39] World Health Organization. Process of translation and adaptation of instruments. https://www.who.int/substance_abuse/research_tools/translation/en/. Accessed 2 April 2019.

[40] Kline RB (2010). Principles and practice of structural equation modeling.

[41] Litzelman DK, Stratos GA, Marriott DJ, Skeff KM (1998). Factorial validation of a widely disseminated educational framework for evaluating clinical teachers. Acad Med.

[42] Cambra-Fierro JJ, Polo-Redondo Y (2008). Creating satisfaction in the demand-supply chain: the buyers’ perspective. Supply Chain Management: An International Journal.

[43] Gonzalez R, Griffin D (2001). Testing parameters in structural equation modeling: every “one” matters. Psychol Methods.

[44] Morasso G, Capelli M, Viterbori P (1999). Psychological and symptom distress in terminal cancer patients with met and unmet needs. J Pain Symptom Manag.

[45] Hasegawa T, Goto N, Matsumoto N (2016). Prevalence of unmet needs and correlated factors in advanced-stage cancer patients receiving rehabilitation. Support Care Cancer.

[46] Osse BH, Vernooij-Dassen MJ, Schadé E (2005). The problems experienced by patients with cancer and their needs for palliative care. Support Care Cancer.

[47] Teunissen SC, de Haes HC, Voest EE (2006). Does age matter in palliative care?. Crit Rev Oncol Hematol.

[48] Houts PS, Harvey HA, Hartz AJ (1988). Unmet needs of persons with cancer in Pennsylvania during the period of terminal care. Cancer.

[49] Hwang SS, Chang VT, Cogswell J (2004). Study of unmet needs in symptomatic veterans with advanced cancer: incidence, independent predictors and unmet needs outcome model. J Pain Symptom Manag.

[50] DeVon HA, Block ME, Moyle-Wright P, Ernst DM, Hayden SJ, Lazzara DJ, Savoy SM, Kostas-Polston E (2007). A psychometric toolbox for testing validity and reliability. J Nurs Scholarsh.

